# Rapid and Sensitive Diagnosis of Drug-Resistant FLT3-F691L Mutation by CRISPR Detection

**DOI:** 10.3389/fmolb.2021.753276

**Published:** 2021-10-25

**Authors:** Yin Liu, Yanling Chen, Shisheng Huang, Xiaodong Ma, Xingxu Huang, Xinjie Wang, Fuling Zhou

**Affiliations:** ^1^ Department of Hematology, Zhongnan Hospital of Wuhan University, Wuhan, China; ^2^ School of Life Science and Technology, ShanghaiTech University, Shanghai, China; ^3^ Key Laboratory of Brain, Cognition and Education Sciences, Institute for Brain Research and Rehabilitation, South China Normal University, Ministry of Education, Guangzhou, China; ^4^ Shenzhen Branch, Guangdong Laboratory of Lingnan Modern Agriculture, Genome Analysis Laboratory of the Ministry of Agriculture and Rural Affairs, Agricultural Genomics Institute at Shenzhen, Chinese Academy of Agricultural Sciencesn, Shenzhen, China

**Keywords:** drug resistance, acute myeloid leukemia, FMS-like tyrosine kinase 3, F691L mutation, CRISPR detection

## Abstract

Sensitive and efficient detection of drug-resistant mutations is essential in cancer precision medicine. In treating acute myeloid leukemia (AML), FLT3 gene F691L mutation shows universal resistance to all currently available FLT3 inhibitors. However, there is no particular detection method for FLT3-F691L. Commonly-used first-generation sequencing (FGS) approaches have low sensitivity, and next-generation sequencing (NGS) is time-consuming. Herein, we developed an accurate and sensitive FLT3-F691L diagnostic method by CRISPR detection. Briefly, the FLT3-691 region is amplified by recombinase polymerase amplification (RPA) and detected by L691-crRNA induced Cas12a reaction, and finally the result can be directly observed under a blue lamp or analyzed by a fluorescence reader. Confirmed by the tests on diluted plasmids and 120 AML patient samples, this method can achieve a sensitivity of 0.1% and complete the whole diagnosis process within 40 min. Potentially, this method will play an important role in point-of-care applications and guidance of AML treatment.

## Introduction

Acute myeloid leukemia (AML), similar to many other cancers, is characterized by various somatically acquired mutations that affect gene functions (Dohner et al., 2021). FMS-like tyrosine kinase 3 (FLT3) gene mutations were observed in approximately 30% of AML cases, including 25% internal tandem duplication (ITD) and 7–10% tyrosine kinase domain (TKD) mutation (Daver et al., 2019). The deep understanding of FLT3 mutations leads to the emergence of multiple FLT3 inhibitors, which are now one of the most promising treatments for AML (Kiyoi et al., 2020). The first-generation FLT3 inhibitors are multi-targeted, include midostaurin, sunitinib, lestaurtinib, sorafenib, and tandutinib. The second-generation FLT3 inhibitors are more selective and effective as single agents, represented by gilteritinib, crenolanib, and quizartinib. Most FLT3 mutated cases are sensitive to at least one of them. However, a “gatekeeper” mutation FLT3-F691L shows universal resistance to all these inhibitors (Eguchi et al., 2020; Smith et al., 2015; Tarver et al., 2020). Therefore, the early detection of FLT3-F691L mutation is of vital importance in avoiding ineffective treatment for AML patients.

Currently-used FLT3-F691L detection methods include first-generation sequencing (FGS) and next-generation sequencing (NGS) approaches, and both have strengths and limitations. FGS is now a well-established technology that can output results within 1 day, but its low sensitivity limits the detection of a mutation rate of less than 10%. On the other hand, NGS is much more sensitive and accurate. However, NGS is too time-consuming (more than 1 week) and not economical. Therefore, a rapid and sensitive method for FLT3-F691L detection is still needed.

Recently, the clustered regularly interspaced short palindromic repeats (CRISPR) based nucleic acid detection was developed, with the advantages of sensitivity, specificity, and rapidity (Li et al., 2019). The core mechanism of CRISPR detection is that CRISPR-associated (Cas) proteins can specifically recognize targeted nucleic acids under the guidance of a single-stranded guide CRISPR RNA (crRNA). The most frequently used Cas proteins are Cas9, Cas12, and Cas13 (Gootenberg et al., 2017; Li et al., 2018a; Li et al., 2018b; Chen et al., 2018; Myhrvold et al., 2018; Zhou et al., 2018; Chen Q. et al., 2020; Wang et al., 2020b). Especially, Cas12a can generate a nonspecific ssDNAase activity upon target recognition, which allows Cas12a to be coupled with an ssDNA fluorescence reporter and construct an ultrasensitive molecular detection method (Wang et al., 2020b). To date, Cas12a-based detection technologies have been used to diagnose various infectious diseases, including SARS-CoV-2, African swine fever virus, Dengue virus, and *Mycobacterium tuberculosis* (Wang et al., 2020a; Curti et al., 2020; Wang et al., 2020c; Ding et al., 2020; Ma et al., 2020; Xu et al., 2020).

Herein, we combined Cas12a-based detection with recombinase polymerase amplification (RPA) to establish a sensitive, accurate and rapid system for FLT3-F691L detection. Potentially, this method will play an important role in point-of-care applications and guidance of AML treatment.

## Materials and Methods

### Patient Samples

Peripheral blood samples of 120 AML patients were collected from the Hematology Department in Zhongnan Hospital of Wuhan University under an approved Institutional Review Board protocol. For DNA template preparation, about 500 µl of peripheral blood was mixed well with four times the volume of the RBC lysis buffer (Biosharp, Hefei, China). After 1 min of lysis, WBCs were precipitated by 1 min of mini centrifugation and then incubated with 150 µl nucleic acid releaser (GenDx, Suzhou, China) at 95°C for 3 min to release genomic DNA. Two microliters of the released DNA were used for subsequent RPA.

### Plasmid and DNA Fragments Preparation

A 630-bp DNA fragment covering the WT FLT3-691 site was amplified by PCR with primers P1 and P2 from a WT patient’s genomic DNA, then cloned into the pGem-T vector (Takara, China). After transformation into *Escherichia coli* DH5α, the T-vec-F691 plasmid was extracted by an AxyPrep Plasmid Miniprep Kit (Axygen, CA, United States). For constructing the FLT3-L691 plasmid, primers P3 and P4 carrying a T > G base mutation were used to amplify the T-vec-F691 plasmid circularly. Then the amplified product was self-ligated and cloned into the pGem-T vector to construct the T-vec-F691 plasmid. For identification of the specificity of crRNAs, 350-bp F691 and L691 DNA fragments were produced by PCR with primers P5 and P6 using T-vec-F691 and FLT3-L691 plasmids as templates. The quantification of the above plasmids and DNA fragments were conducted by a Nanodrop2000 (Thermo Fisher Scientific, MA, United States). The sequence information of PCR primers are listed in Supplementary Table S1.

### RPA

The RPA assay was conducted by a GenDx ERA Kit (Suzhou GenDx Biotech, China). Briefly, the 50 µl RPA system included 2 µl DNA template, 2.5 µl forward primer (10 nM), 2.5 µl reverse primer (10 nM), 11 µl ERA basic buffer, 20 µl reaction buffer, 2 µl activator, and supplementary ddH2O. After sufficient mixing, the mixture was incubated at 37°C for 15 min. Five microliters of the RPA product were transferred to subsequent Cas12a reaction. The RPA primers are listed in Supplementary Table S1.

### Cas12a Fluorescence Reaction

Cas12a fluorescence reaction was conducted according to a previous description (Wang et al., 2020a). The 20 µl reaction mixture included 200 ng Cas12a, 0.1 pmol crRNA, 2 µl 10 × NEBuffer 3.1 (NEB, MA, United States), 1 µl RNase inhibitor (Novoprotein, China), 25 pmol ssDNA-FQ reporter (Genewiz, NJ, America), an appropriate amount of DNA fragments or 5 µl RPA product, and supplementary ddH2O. Among these components, crRNAs were directly synthesized by GenScript (Nanjing, China). And the sequence information of F691-crRNA and L691-crRNA are listed in Supplementary Table S2. Cas12a protein was produced as described previously (Creutzburg et al., 2020). The mixed Cas12a reaction system was incubated at 37°C for 15 min, and then the result was directly observed under a 485-nm blue lamp (Sangon, Shanghai, China). Fluorescence kinetics were monitored using a monochromator with excitation at 485 nm and emission at 520 nm.

### FGS and NGS

For FGS detection, 30 µl PCR products or 30 µl RPA products were purified by the AxyPrep PCR Clean-up Kit and quantified by the Nanodrop2000. Approximately 200 ng DNA products were sent to FGS conducted by Tsingke (Beijing, China). Different barcoded primers were designed and synthesized for the PCR amplification of the FLT3-691 region of genomic DNA samples for NGS detection. The amplified products were then purified and mixed equally for NGS detection by the Illumina NextSeq 500 (2 × 150) platform at the CAS-MPG Partner Institute for Computational Biology Omics Core, Shanghai, China. The PCR primers for the preparation of NGS samples are listed in Supplementary Table S3.

### Statistical Analysis

We have repeated all experiments three times. Statistical analysis was performed by GraphPad Prism software version 8.0. Unpaired two-tailed Student’s t-test was used for comparison between two groups. Quantitative data are expressed as mean value ± SE. *p < 0.05, **p < 0.01, ***p < 0.001, ****p < 0.0001; ns, no significance.

## Results

### Development of the CRISPR Detection System for FLT3-F691L Diagnosis

**FIGURE 1:**
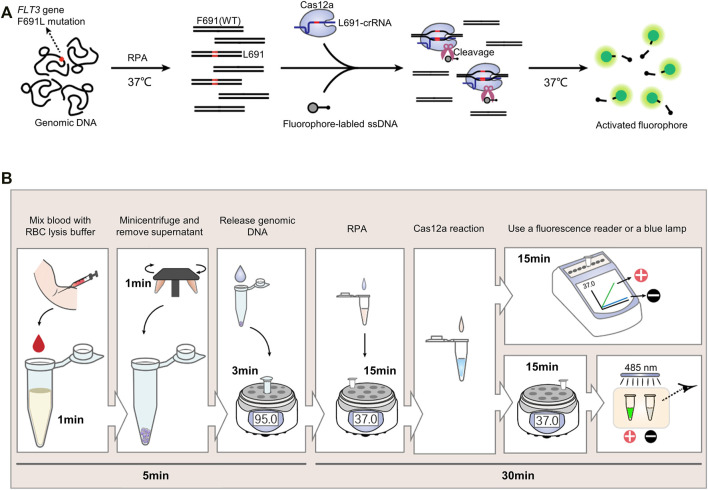
The CRISPR detection system for FLT3-F691L diagnosis. **(A)** Schematic Diagram of the CRISPR detection method for FLT3-F691L diagnosis. **(B)** Flowcharts of the whole process from drawing blood to receiving the result in the detection of FLT3-F691L mutation by CRISPR detection.

Commonly-used CRISPR detection includes two steps, isothermal amplification and Cas12a fluorescence reaction. Recombinase polymerase amplification (RPA) is a highly selective and efficient isothermal amplification technique, which allows the amplification of single-digit DNA target copies under 37–42°C (Lobato and O’Sullivan, 2018). In this study, RPA was adopted to amplify the FLT3-691 region of genomic DNA. Then RPA product was detected by L691-crRNA induced Cas12a reaction, wherein fluorophore-labeled ssDNA existed. Upon recognizing F691L mutant amplicons, Cas12a will generate nonspecific ssDNAase activity to cleave fluorophore-labeled ssDNA and release fluorescence signal (Figure 1A).

We developed a complete diagnosis process from drawing blood to receiving the clinical application of this CRISPR detection system. First, the blood drawn from the patient was mixed with red blood cell (RBC) lysis buffer for 1 min. Then 1 min of centrifugation was adopted to obtain white blood cell (WBC) precipitation. Next, the genomic DNA of these WBCs was released by a nucleic acid releaser under 95°C for 3 min. Then, the DNA sample was amplified by RPA under 37°C for 15 min, and detected by L691-crRNA induced Cas12a reaction under 37°C for 15 min. Finally, the result can be analyzed by a fluorescence reader or directly observed by naked eyes under a blue lamp. Increased fluorescence intensity and green fluorescence signal indicate a F691L positive result. On the contrary, unchanged fluorescence intensity and no green fluorescence signal suggest a F691L negative result (Figure 1B). Thus, the whole diagnosis can be completed within 40 min, without the need for large equipment or expensive reagent.

### Identification of the Specificity of crRNA for F691L Detection

**FIGURE 2:**
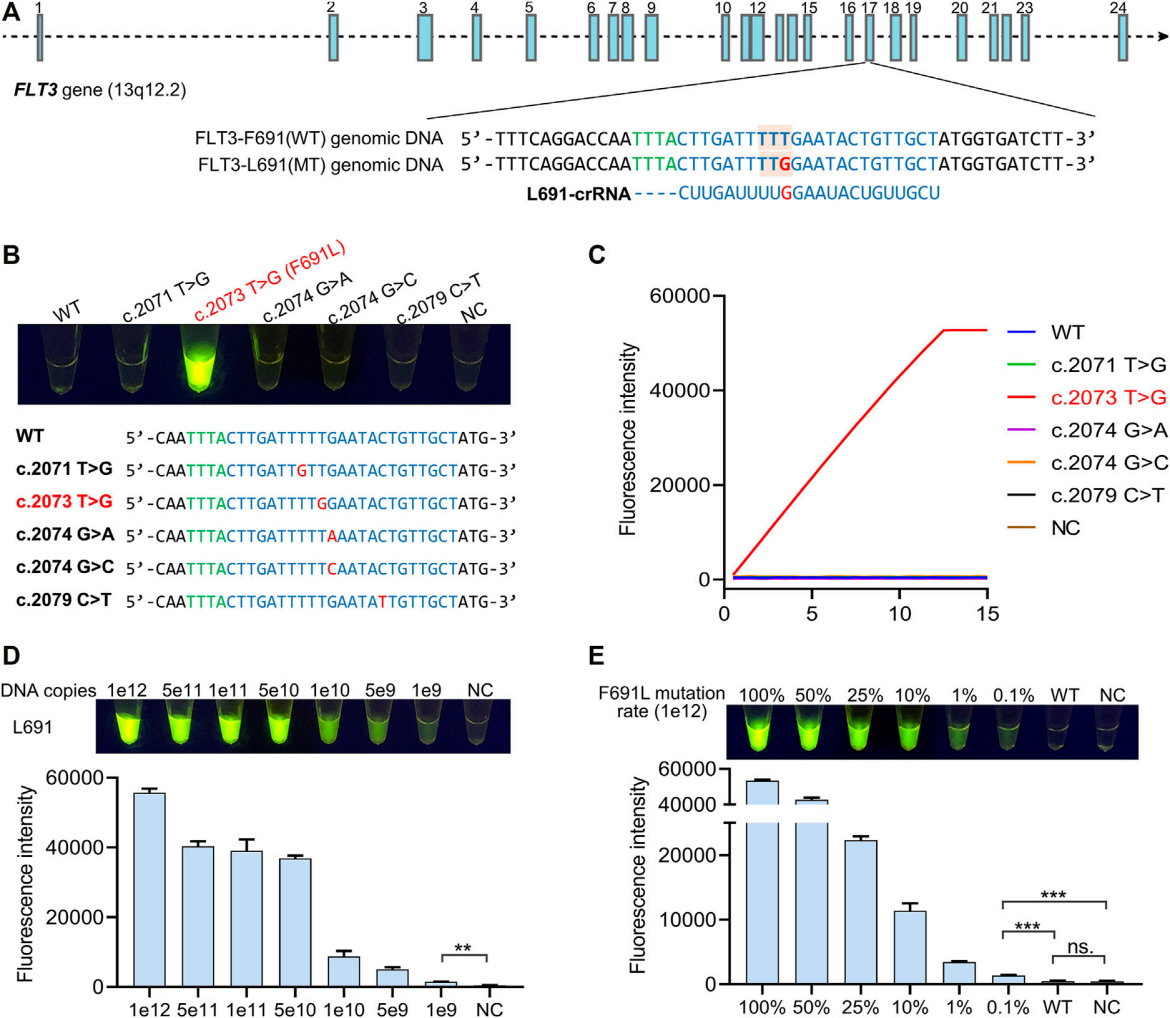
Identification of the specificity of crRNA for F691L detection. **(A)** FLT3 gene structure diagram, sequences of WT and mutant genomic DNA, and L691-crRNA. Blue boxes show the 24 exons of FLT3 gene. FLT3-691 region in exon 17 is partially magnified. Green bases represent the specific protospacer adjacent motif (PAM) of Cas12a. The amino acid codon of 691 site is marked by orange boxes, and the T > G mutation is colored in red. **(B)** The naked-eye result of L691-crRNA induced Cas12a reaction in the detection of 1e12 copies of WT, F691L, and other four mutation DNA fragments. The reaction is under 37°C for 15 min. NC, negative control. The sequences of these samples are shown below. **(C)** time-course analysis of **(B)**. **(D)** The naked-eye result of L691-crRNA induced Cas12a reaction in the detection of gradient copies of L691 DNA fragments. The reaction is under 37°C for 15 min. The statistical chart is shown below. **(E)** The naked-eye result of L691-crRNA induced Cas12a reaction in the detection of 1e12 copies of DNA fragments with different F691L mutation rates. The reaction is under 37°C for 15 min. The statistical chart is shown below.

The high specificity of the Cas12a reaction relies on a specific crRNA, which only recognizes target DNA while ignores non-targets. For the detection of mutation-type (MT) FLT3-L691 (c. TTG) from wild-type (WT) FLT3-F691 (c. TTT), we designed a crRNA with a sequence complementary to L691 genomic DNA (Figure 2A). To identify the specificity of L691-crRNA, we used L691-crRNA induced Cas12a reaction to detect 1e12 copies of WT and L691 DNA fragments, as well as other four mutations in the detection region, which were recorded in the catalogue of somatic mutations in cancer (COSMIC) database (Supplementary Table S4). After 15 min reaction under 37°C, the L691 sample showed a strong fluorescence signal, and no fluorescence was observed in WT and other mutation samples. These results confirmed the high specificity of L691-crRNA (Figures 2B,C). Then gradient copies (1e12 ∼ 1e9) of L691 DNA fragments were used to identify the sensitivity of L691-crRNA induced Cas12a reaction. The result showed that as low as 1e9 copies of L691 DNA fragments could be detected (Figure 2D). Finally, we mixed 1e12 copies of F691 and L691 DNA fragments with different proportions to simulate mutation rates (100, 50, 25, 10, 1, 0.1%, and WT). As expected, the fluorescence signals gradually decreased with mutation rates. A significant difference could be observed between 0.1% sample and the negative control, while no significant difference was found between WT sample and the negative control. Together, these results indicated that L691-crRNA is highly specific for the detection of FLT3-F691L mutation (Figure 2E). In the same way, we also designed an F691-crRNA for the detection of WT FLT3-F691 (Supplementary Figrue S1).

### RPA Primer Screening for Highly Sensitive Detection

**FIGURE 3:**
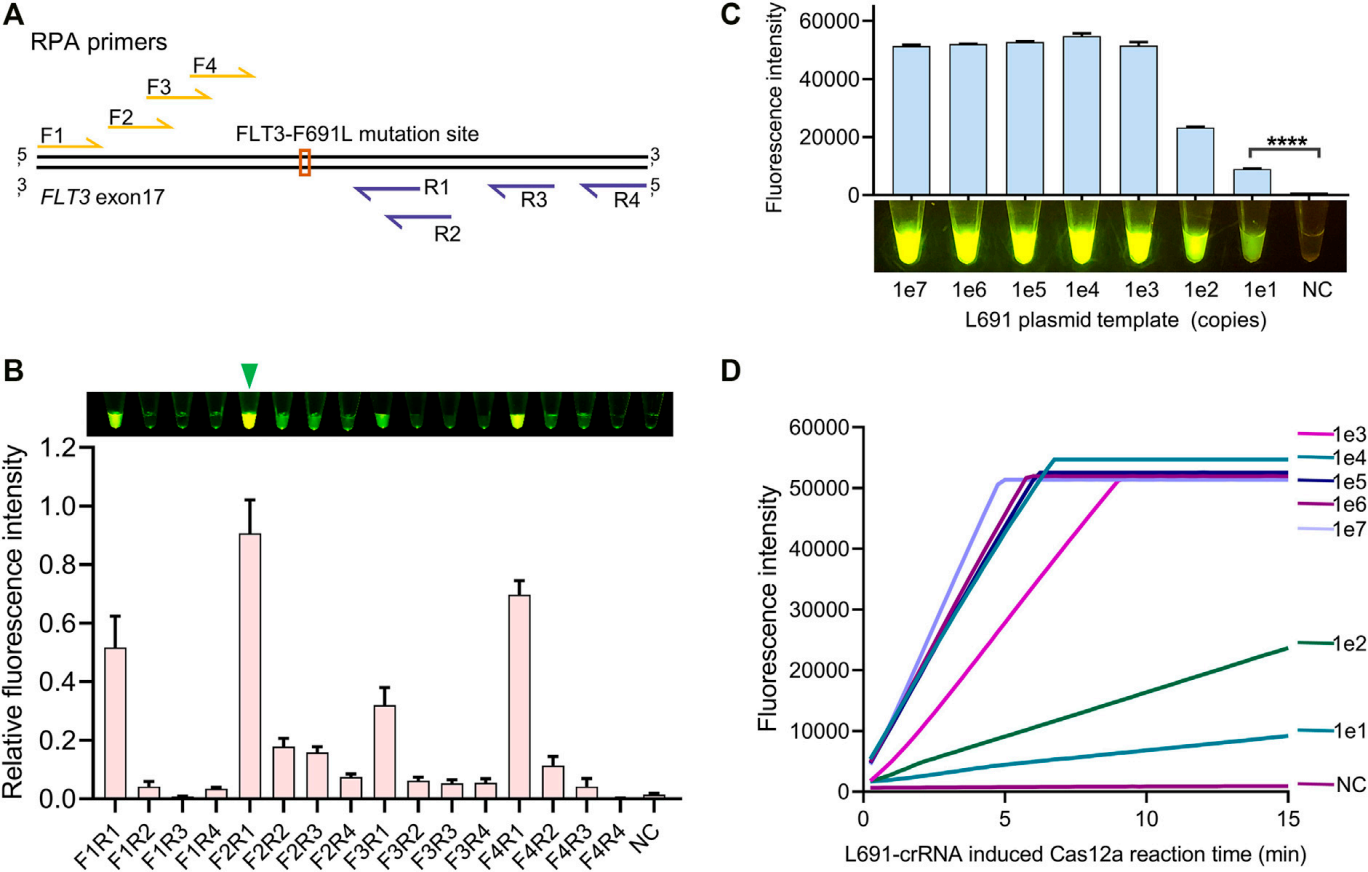
RPA primer screen for highly sensitive detection. **(A)** Relative locations of RPA primers to the FLT3-691 site. Forward and reverse primers are colored in yellow and purple, relatively. **(B)** Comparison of amplification efficiency between different RPA primer pairs. The naked-eye results and fluorescence intensity results are shown above and below, respectively. The relative fluorescence intensity is normalized by setting the highest value to 1. A green triangle points out the most efficient F2R1 pair. **(C)** The identification of the amplification capacity of F2R1-mediated RPA using 1e7 ∼ 1e1 gradient copies of L691 plasmid templates. This assay was conducted by 15 min of RPA and 15 min of L691-crRNA induced Cas12a reaction, both under 37°C. The fluorescence intensity results and the naked-eye results are shown above and below, respectively. **(D)** Time-course analysis of **(C)**.

To achieve an ultrasensitive detection of FLT3-F691L mutation, a highly efficient amplification is needed to produce enough target copies. According to the design principles of RPA primers, we designed four forward primers RPA-F1 ∼ 4 and four reverse primers RPA-R1 ∼ 4 for the amplification of the FLT3-691 region (Figure 3A). Then 16 primer pairs were generated by pairwise collocation between forward and reverse primers, and they were tested to screen out the most efficient pair. In this test, 1e2 copies of L691 plasmid templates were used to simulate the genomic DNA, and the RPA products were detected by L691-crRNA induced Cas12a reaction. After 15 min of RPA with different primer pairs and 15 min of Cas12a reaction, the strongest fluorescence signal was observed in the F2R1 pair, indicating that F2R1 is most efficient to amplify the FLT3-691 region (Figure 3B). In order to analyze its amplification capacity more specifically, we used F2R1-mediated RPA to amplify 1e7 ∼ 1e1 gradient copies of L691 plasmid templates. After L691-crRNA induced Cas12a reaction, the results showed that as low as 1e1 copies of L691 plasmids could be successfully detected (Figures 3C,D and Supplementary Figure S2). Together, using 15 min of F2R1-mediated RPA and 15 min of L691-crRNA induced Cas12a reaction, the FLT3-F691L detection system can achieve detection of nearly single-digit copies of pure L691 DNA templates.

### Identification of the Sensitivity of FLT3-F691L Detection System

**FIGURE 4:**
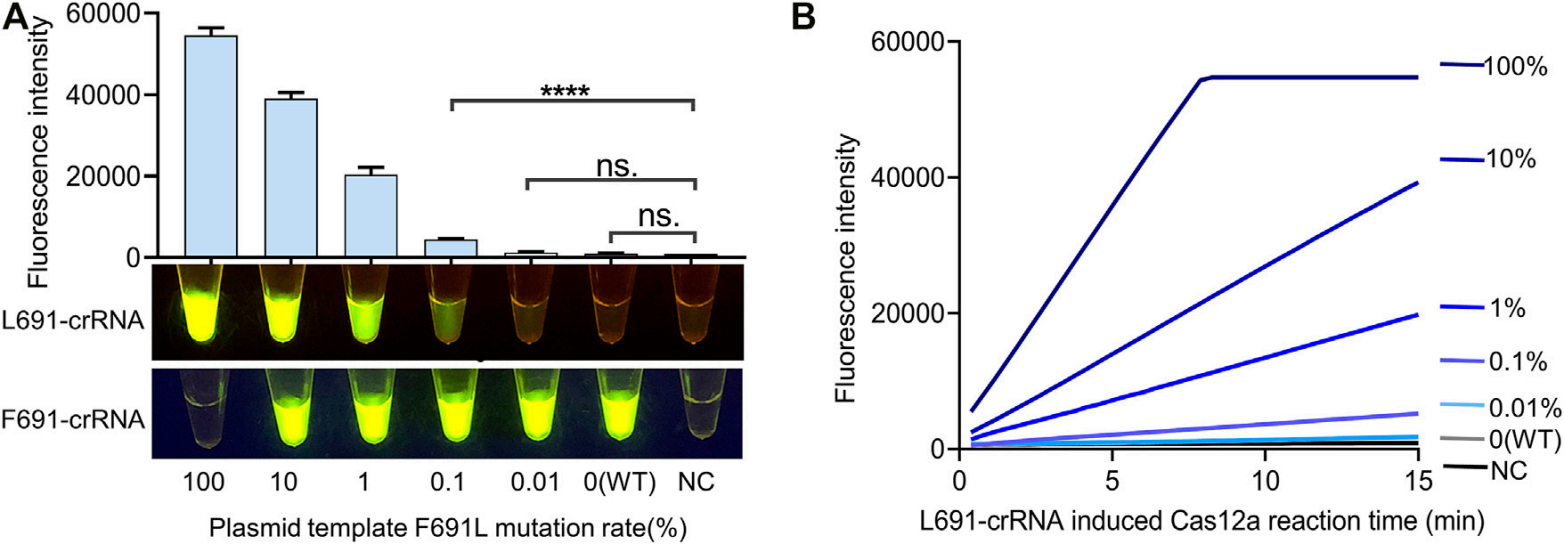
Identification of the sensitivity of FLT3-F691L detection system. **(A)** Sensitivity assay of the CRISPR detection system for FLT3-F691L mutation. Plasmids with gradient F691L mutation rates are used as tested samples. The detection was conducted by 15 min of F2R1-mediated RPA and 15 min of L691-crRNA induced Cas12a reaction, both under 37°C. Final fluorescence intensity results and naked-eye results are shown above and below, respectively. The naked-eye results of F691-crRNA induced Cas12a reaction are also shown below. **(B)** Time-course analysis of the L691-crRNA induced Cas12a reaction in **(A)**.

Detection of low-abundance FLT3-F691L mutation may help early clinical diagnosis and avoid ineffective treatment. In order to define the sensitivity of the FLT3-F691L detection system, 1e5 copies of plasmids with gradient F691L mutation rates were used as tested samples. The mutation rates included 100% (pure L691), 10, 1, 0.1, 0.01%, and 0 (WT, pure F691), generated by different proportions of L691 and F691 plasmids. These samples were amplified by RPA and then detected by L691-induced Cas12a reaction as mentioned above. The results showed that the detection system could achieve a sensitivity of 0.1%, which means detecting 100 copies of L691 templates among 99,900 copies of F691 templates. Besides, there is no significant difference between WT and NC samples, which further confirmed the high specificity of this detection system (Figures 4A,B and Supplementary Figure S3). The RPA products of these samples were also detected by F691-crRNA to show the amplification of WT templates (Figure 4A). To analyze the fluorescence change more clearly, we use a fluorescence reader to record the time course of the L691-crRNA induced Cas12a reaction. The gradually increased fluorescence intensity in 0.1% sample was observed, compared with unchanged fluorescence intensity in 0.01%, WT, and NC samples. Together, these results indicated that our FLT3-F691L detection system could achieve a sensitivity of 0.1% under 1e5 copies of templates, which is a 100-fold improvement compared with FGS.

### Screening of AML Samples for FLT3-F691L by CRISPR Detection

**FIGURE 5:**
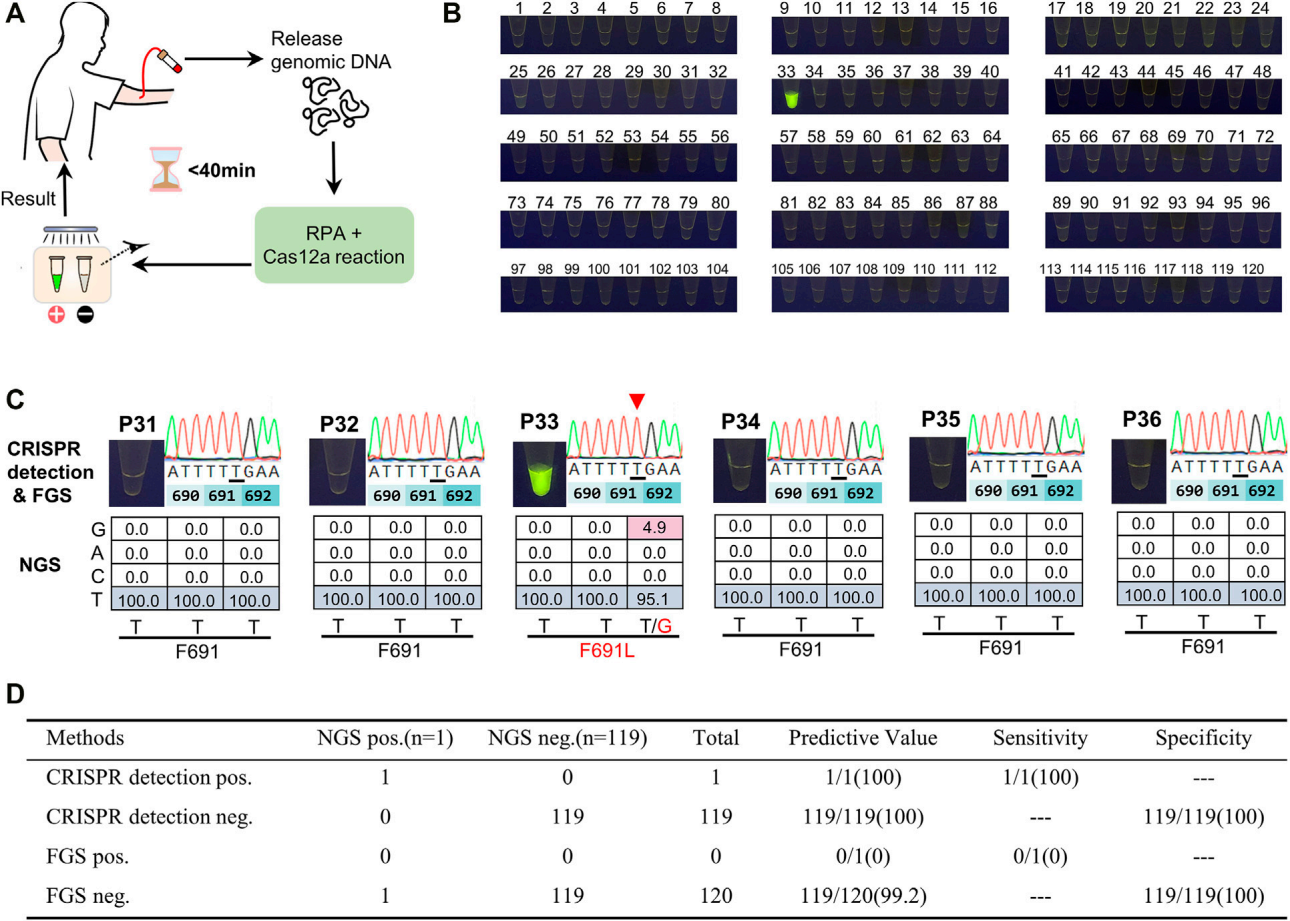
Screening of AML samples for FLT3-F691L mutation by CRISPR detection. **(A)** Schematic diagram of the whole mutation detection process. **(B)** Detection results of 120 AML patients using CRISPR detection for FLT3-F691L mutation. P33 is a FLT3-F691L positive sample. **(C)** The CRISPR detection, FGS, and NGS results of P33 and other five FLT3-F691L negative patients. In FGS results, mutation base is underlined and pointed out by a red triangle. In NGS results, WT and mutation bases are colored in blue a red, respectively. **(D)** Statistical table of the sensitivity and specificity of CRISPR detection compared with FGS, using NGS as a standard reference.

We then applied CRISPR detection to screen AML patients for FLT3-F691L positivity. Briefly, blood samples of 120 AML patients were drawn and the genomic DNA was released, then the DNA templates were used to RPA and L691-induced Cas12a reaction, and finally, the results were observed by naked eyes under a blue lamp. The whole detection was completed within 40 min (Figure 5A). Out of the 120 samples, represented by P1∼120, we found only one FLT3-F691L positive case P33 (Figure 5B and Supplementary Figure S4–S6). This is consistent with the low frequency of FLT3-F691L mutation in AML populations. The NGS data of the 120 patients confirmed the CRISPR detection results, wherein P33 had a F691L mutation rate of 4.9%. However, this sample was not screened out using FGS, further indicating the low sensitivity of FGS (Figure 5C). Using NGS as the gold standard, we compared CRISPR detection with FGS to detect the 120 AML patients. The statistics showed that although CRISPR detection and FGS both performed a 100% specificity in this test, CRISPR detection displayed a much higher sensitivity than FGS (100 vs. 0%) (Figure 5D). Together, these results verified the high efficiency, specificity, and sensitivity of the CRISPR detection method for FLT3-F691L.

## Discussion

In the past 2 decades since FLT3-ITD and TKD mutations were identified in acute myeloid leukemia (Nakao et al., 1996; Yamamoto et al., 2001), researchers have continued unremitting exploration on FLT3-targeted therapies. Happily, three FLT3 inhibitors including midostaurin, gilteritinib and quizartinib, have been finally approved for clinical use in 2017–2019 (Levis, 2017). It means that more than a quarter of AML patients could have a chance to improve their survival. However, the development of drug resistance has challenged the application of FLT3 inhibitors, with drug-resistant point mutations as the most important cause. Although rarely observed in AML patients, FLT3 gene F691L mutation shows universal resistance to all available FLT3 inhibitors. Therefore, F691L mutation becomes a crucial detection item for treatment decisions (Albers et al., 2013; Smith et al., 2015; Yamaura et al., 2018; Wang et al., 2021). Furthermore, earlier identification of drug-resistant mutations will help better understand the progression of the disease, and enable properly targeted treatment and more durable remissions for patients.

Although FGS and NGS have been used in clinical molecular diagnosis, they both have some drawbacks. FGS is relatively rapid but with low sensitivity, while NGS is sensitive but time-consuming and not economical. In this study, we developed an efficient, accurate, and sensitive method for FLT3-F691L detection. This method is based on RPA and Cas12a fluorescence assay; both are operated under 37°C, without the need of large equipment and expensive reagent. Using this method, we achieved a sensitivity of 0.1% in the detection of 1e5 copies of plasmid templates.

Moreover, we used this CRISPR detection to test 120 AML patient samples for clinical use. For the low incidence of FLT3-F691L mutation, only one F691L-mutated patient sample was screened out, which is missed by FGS detection. This positive sample and other 119 negative samples were further confirmed by NGS methods. These results indicate the advantages of our CRISPR detection method compared with FGS. Moreover, the CRISPR detection method can complete the clinical diagnosis of FLT3-F691L within 40 min, which is much more efficient than FGS and NGS. However, due to its high sensitivity, CRISPR detection should be carefully operated in a spotless environment to avoid DNA contamination or cross-contamination among samples. In future work, to reduce the cross contamination and simplify the operation, there are some ways to try, including adding the Cas12a reaction system to the lid of the RPA tube before sealing and one-pot RPA-Cas12a detection approaches (Chen Y. et al., 2020; Feng et al., 2021; Sun et al., 2021).

In conclusion, our highly efficient and sensitive CRISPR detection provides a promising way for the clinical diagnosis of drug-resistant FLT3-F691L mutation. This method will play an important role in early screening, point-of-care and therapy options in AML treatment.

## Data Availability

The raw data supporting the conclusion of this article will be made available by the authors, without undue reservation.
