# Supplementary material for: Rapid and Sensitive Diagnosis of Drug-Resistant FLT3-F691L Mutation by CRISPR Detection
Source: Front Mol Biosci. 2021 Oct 25;8:753276. doi: 10.3389/fmolb.2021.753276 (PMC8574994; doi:10.3389/fmolb.2021.753276)
Supplement: Supplementary file 1 [file DataSheet1.PDF]

Supplementary Material

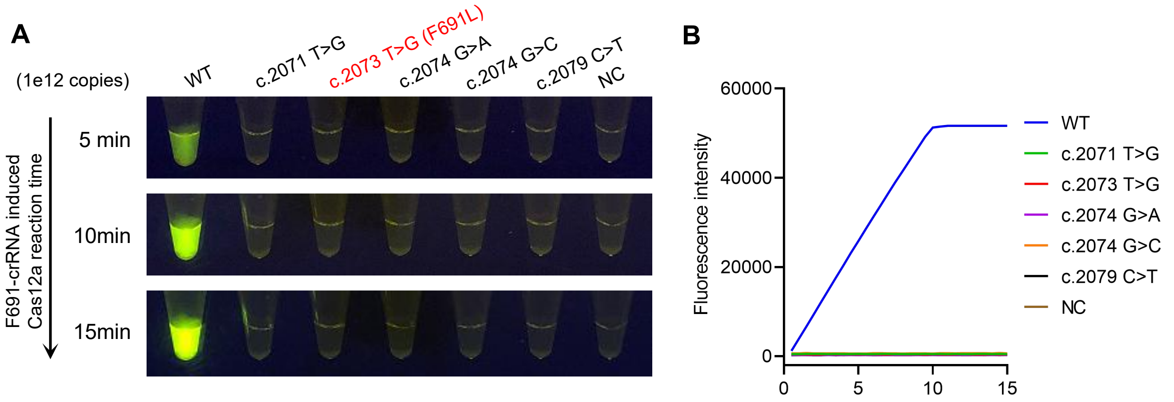

FIGURE S1 | (A) Naked-eye results and **(B)** time-course analysis of F691-crRNA induced Cas12a detection of 1e12 copies of DNA fragments of WT, F691L, and other four possible mutations.

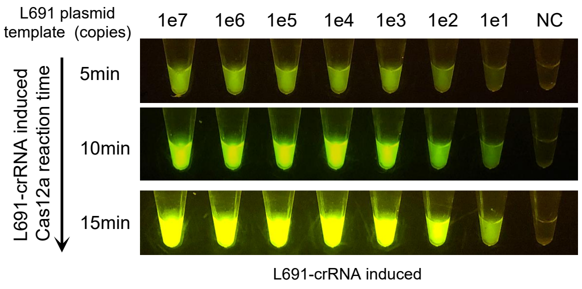

FIGURE S2 | Amplification capacity test of F2R1-mediated RPA. The RPA products were detected by 15 min of L691-crRNA induced Cas12a reaction. The naked-eye results at three time points, 5 min, 10 min, and 15 min were recorded.

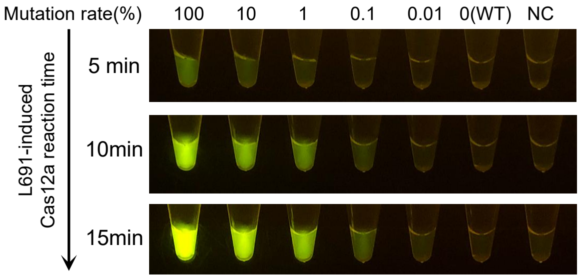

FIGURE S3 | Sensitivity assay of CRISPR detection for FLT3-F691L mutation. The RPA products were detected by 15 min of L691-crRNA induced Cas12a reaction. The naked-eye results at three time points, 5 min, 10 min, and 15 min were recorded.

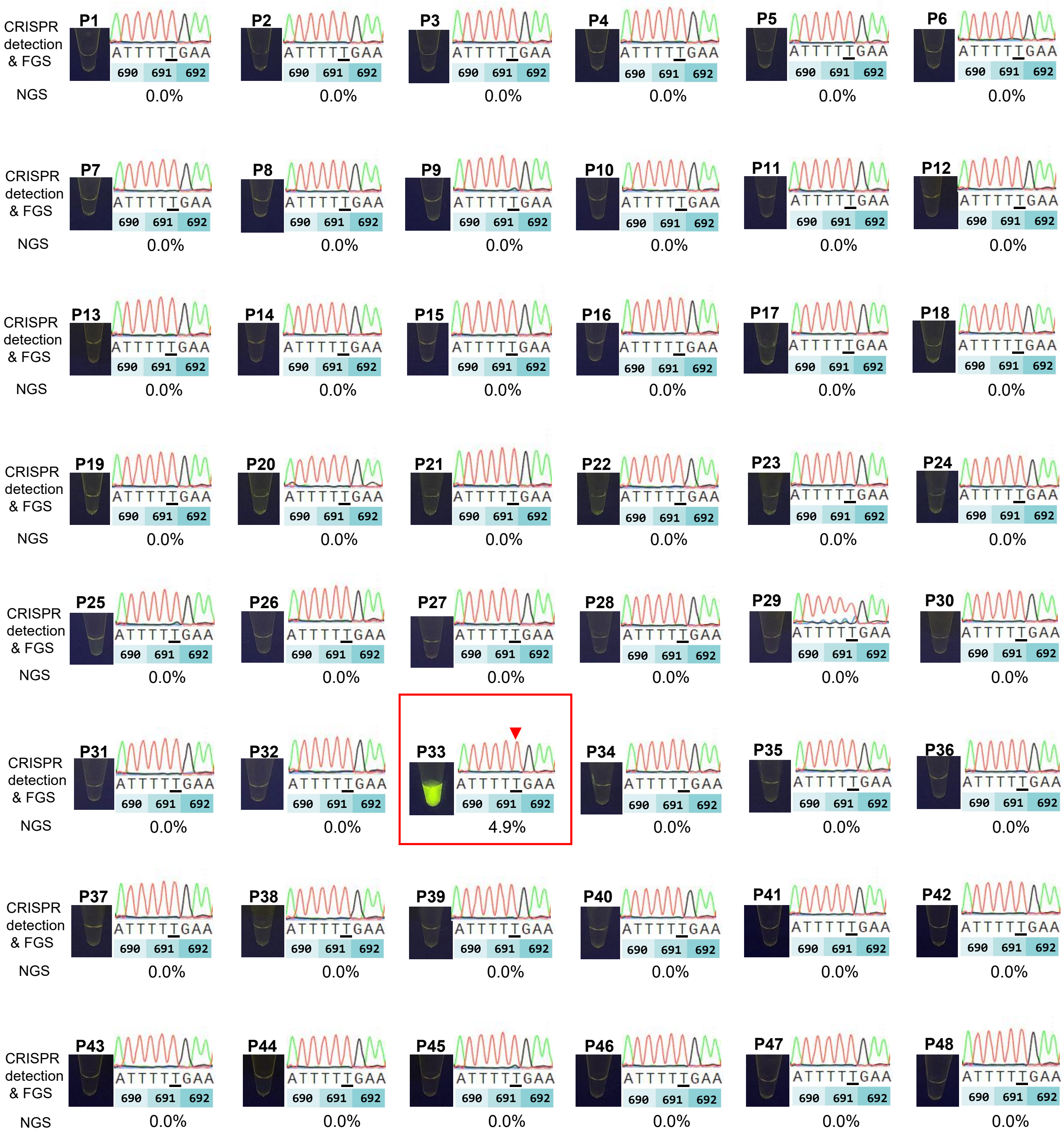

FIGURE S4 | CRISPR detection, FGS and NGS results of Patient 1~48 for FLT3-F691L screening. The positive sample P33 is marked by a red box, and the mutated T > G base is pointed out by a red triangle. The numbers 690, 691, and 692 represent the amino acid codon sites. And the percentage numbers showed the NGS results of TTT > TTG mutation rates.

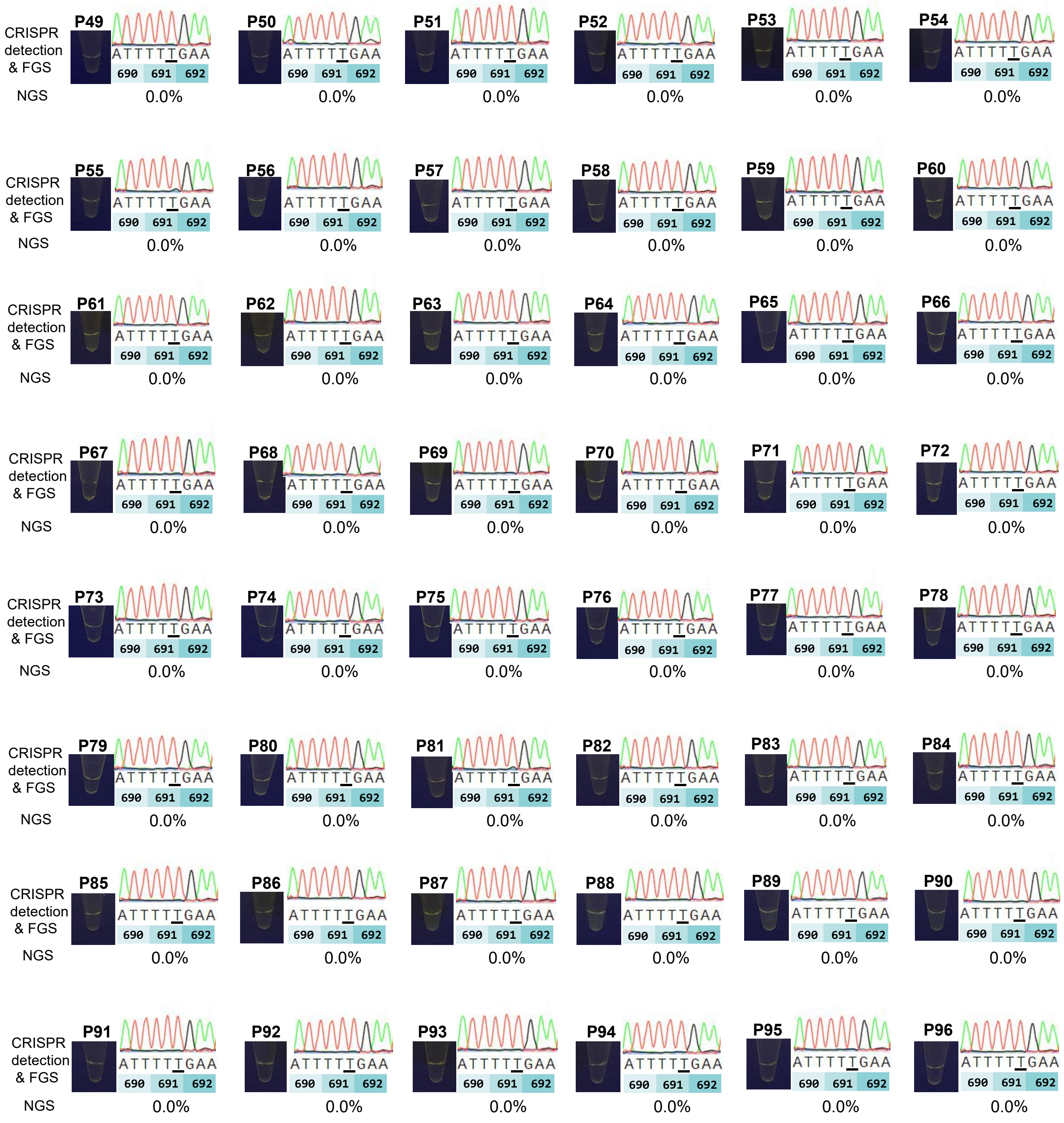

FIGURE S5 | CRISPR detection, FGS and NGS results of Patient 49 ~ 96 for FLT3-F691L screening. The numbers 690, 691, and 692 represent the amino acid codon sites. And the percentage numbers showed the NGS results of TTT > TTG mutation rates.

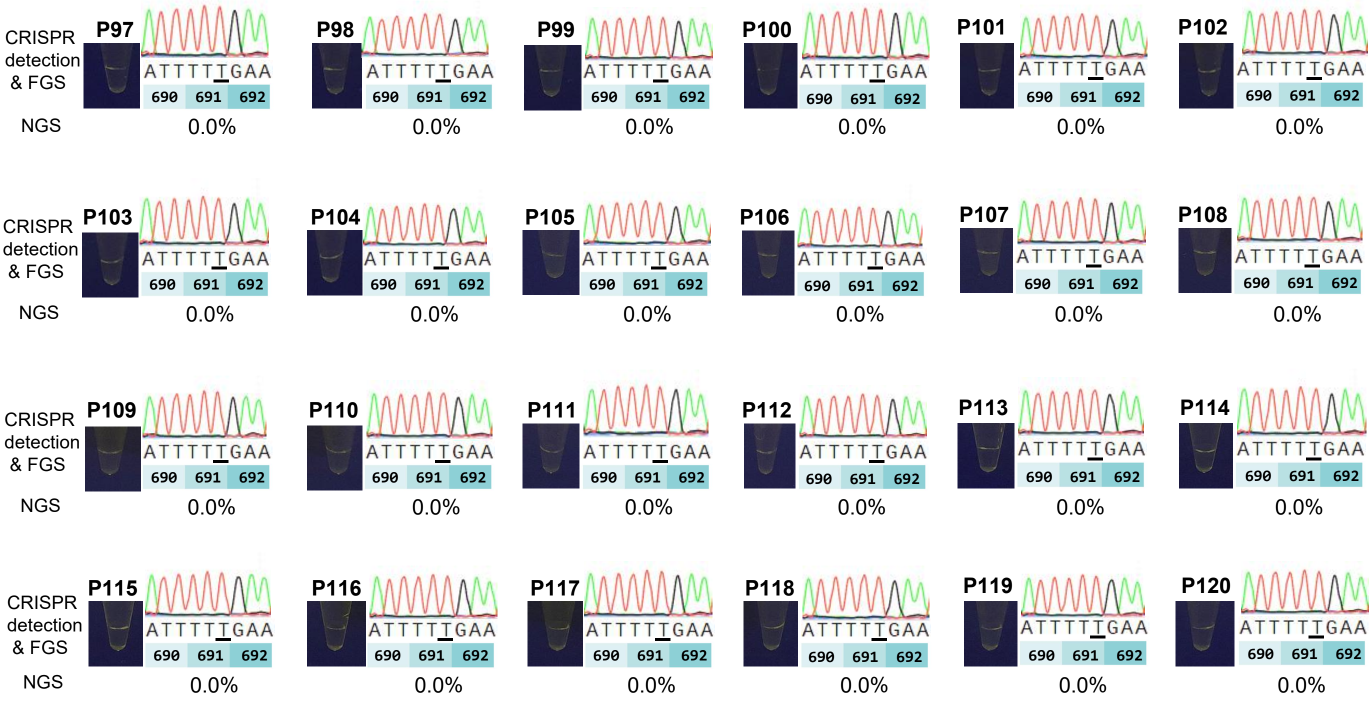

FIGURE S6 | CRISPR detection, FGS and NGS results of Patient 97 ~ 120 for FLT3-F691L screening. The numbers 690, 691, and 692 represent the amino acid codon sites. And the percentage numbers showed the NGS results of TTT > TTG mutation rates.

TABLE S1 PCR and RPA primer sequences

ID	Name	Sequence
P1	FLT3-FLT3-691-630bp-For	TGGTGAGGCCACACATACAAA
P2	FLT3-FLT3-691-630bp-Rev	GGCATGGGTGGAGAGAGACTA
P3	T-vec-FLT3-691L-For	CTTGATTTTGGAATACTGTTGCTATG
P4	T-vec-FLT3-691L-Rev	CAGTATTCCAAAATCAAGTAAATTGG
P5	FLT3-691-350bp-For	ATAAGAAGAGCTAGGCTCAG
P6	FLT3-691-350bp-Rev	GCCTACGTTCTATGATGTG
P7	FLT3-691-RPA-F1	TCCTAAGAGTATGTTGTCTGCTACATAGAC
P8	FLT3-691-RPA-F2	TCTGAAATAACAGTTTGCTTTGTGTATGCC
P9	FLT3-691-RPA-F3	TTTGCTTTGTGTATGCCTATAATTGAAACTG
P10	FLT3-691-RPA-F4	CCTATAATTGAAACTGTAACTATTTCAGGACC
P11	FLT3-691-RPA-R1	TTAGATAGTTGAGAAGATCACCATAGCAACAG
P12	FLT3-691-RPA-R2	TTTACTTCTTAGATAGTTGAGAAGATCACC
P13	FLT3-691-RPA-R3	TTCCTTGAAAATCTCTGTCCAAGTCCTGTG
P14	FLT3-691-RPA-R4	ACCTGGAATTTGGATGTGATTGGAAAGTGG

* Mutated bases are colored in red.

TABLE S2 crRNA sequences

Name	Sequence
FLT3-F691-crRNA	UAAUUUCUACUAAGUGUAGAUCUUGAUUUUUGAAUACUGUUGCU
FLT3-L691-crRNA	UAAUUUCUACUAAGUGUAGAUCUUGAUUUUGGAAUACUGUUGCU

* Target sequences and the mutated base are colored in blue and red, respectively.

TABLE S3 NGS primer sequences

Name	Sequence
FLT3-691-DSF1	ATCACGGTACTGTCCCCAAGTCAGCA
FLT3-691-DSF2	CGATGTGTACTGTCCCCAAGTCAGCA
FLT3-691-DSF3	TTAGGCAGGTACTGTCCCCAAGTCAGCA
FLT3-691-DSF4	TGACCAGGTACTGTCCCCAAGTCAGCA
FLT3-691-DSF5	ACAGTGCTGTACTGTCCCCAAGTCAGCA
FLT3-691-DSF6	GCCAATGTACTGTCCCCAAGTCAGCA
FLT3-691-DSF7	CAGATCTGTACTGTCCCCAAGTCAGCA
FLT3-691-DSF8	ACTTGAAAGTACTGTCCCCAAGTCAGCA
FLT3-691-DSF9	GATCAGGGTACTGTCCCCAAGTCAGCA
FLT3-691-DSF10	TAGCTTCCGTACTGTCCCCAAGTCAGCA
FLT3-691-DSF11	GGCTACGTACTGTCCCCAAGTCAGCA
FLT3-691-DSF12	CTTGTAGTACTGTCCCCAAGTCAGCA
FLT3-691-DSF13	AGTCAAGTACTGTCCCCAAGTCAGCA
FLT3-691-DSF14	AGTTCCTGTACTGTCCCCAAGTCAGCA
FLT3-691-DSF15	ATGTCAGCGTACTGTCCCCAAGTCAGCA
FLT3-691-DSF16	CCGTCCATGTACTGTCCCCAAGTCAGCA
FLT3-691-DSF17	GTAGAGCGTACTGTCCCCAAGTCAGCA
FLT3-691-DSF18	GTCCGCAGTACTGTCCCCAAGTCAGCA
FLT3-691-DSF19	GTGAAATCGTACTGTCCCCAAGTCAGCA
FLT3-691-DSF20	GTGGCCGTACTGTCCCCAAGTCAGCA
FLT3-691-DSF21	GTTTCGGTACTGTCCCCAAGTCAGCA
FLT3-691-DSF22	CGTACGGGTACTGTCCCCAAGTCAGCA
FLT3-691-DSF23	GAGTGGAGGTACTGTCCCCAAGTCAGCA
FLT3-691-DSF24	GGTAGCAGTACTGTCCCCAAGTCAGCA
FLT3-691-DSF25	ACTGATGGTACTGTCCCCAAGTCAGCA
FLT3-691-DSF26	ATGAGCCAGTACTGTCCCCAAGTCAGCA
FLT3-691-DSF27	ATTCCTGTACTGTCCCCAAGTCAGCA
FLT3-691-DSF28	CACCGGCAGTACTGTCCCCAAGTCAGCA
FLT3-691-DFR	AATCTCTGTCCAAGTCCTGTGA

TABLE S4 Mutations in L691-crRNA detection region in COSMIC database.

Gene	Genomic mutation ID	AA mutation	CDS mutation	Sample name	PMID
FLT3	COSV54067517	p.F691V	c.2071T>G	P-0005824-T01-IM5	28481359
FLT3	COSV54050694	p.F691L	c.2073T>G	1863609	22504184
FLT3	COSV54050694	p.F691L	c.2073T>G	1863611	22504184
FLT3	COSV54050694	p.F691L	c.2073T>G	1863619	22504184
FLT3	COSV54050694	p.F691L	c.2073T>G	2531201	23392356
FLT3	COSV54050694	p.F691L	c.2073T>G	2531276	23969938
FLT3	COSV54050694	p.F691L	c.2073T>G	2531279	23969938
FLT3	COSV54050694	p.F691L	c.2073T>G	2531281	23969938
FLT3	COSV105022020	p.E692K	c.2074G>A	5-GE077-T1	26950094
FLT3	COSV99609505	p.E692Q	c.2074G>C	2790140	29506494
FLT3	COSV54070221	p.Y693=	c.2079C>T	HT115	24755471

* COSMIC database website: https://cancer.sanger.ac.uk/cosmic.
